# *Salmonella* Typhimurium DT193 and DT99 are present in great and blue tits in Flanders, Belgium

**DOI:** 10.1371/journal.pone.0187640

**Published:** 2017-11-07

**Authors:** R. Boonyarittichaikij, E. Verbrugghe, D. Dekeukeleire, R. De Beelde, L. O. Rouffaer, R. Haesendonck, D. Strubbe, W. Mattheus, S. Bertrand, F. Pasmans, D. Bonte, K. Verheyen, L. Lens, A. Martel

**Affiliations:** 1 Department of Pathology, Bacteriology and Avian Diseases, Faculty of Veterinary Medicine, Ghent University, Salisburylaan 133, Merelbeke, Belgium; 2 Department of Clinical Sciences and Public Health, Faculty of Veterinary Science, Mahidol University, Phuttamonthon, Nakhon Pathom, Thailand; 3 Terrestrial Ecology Unit, Department of Biology, Ghent University, K.L. Ledeganckstraat 35, Gent, Belgium; 4 Forest & Nature Laboratory, Department Forest and Water Management, Ghent University, Gontrode, Belgium; 5 Center for Macroecology, Evolution and Climate, Natural History Museum of Denmark, University of Copenhagen, Universitetsparken 15, Copenhagen, Denmark; 6 NRC Salmonella and Shigella Bacterial Diseases Division, Scientific Institute of Public Health, Juliette Wytsmanstraat 14, Brussels, Belgium; Laboratoire National de Santé, LUXEMBOURG

## Abstract

Endemic infections with the common avian pathogen *Salmonella enterica* subspecies *enterica* serovar Typhimurium (*Salmonella* Typhimurium) may incur a significant cost on the host population. In this study, we determined the potential of endemic *Salmonella* infections to reduce the reproductive success of blue (*Cyanistes caeruleus*) and great (*Parus major*) tits by correlating eggshell infection with reproductive parameters. The fifth egg of each clutch was collected from nest boxes in 19 deciduous forest fragments. Out of the 101 sampled eggs, 7 *Salmonella* Typhimurium isolates were recovered. The low bacterial prevalence was reflected by a similarly low serological prevalence in the fledglings. In this study with a relatively small sample size, presence of *Salmonella* did not affect reproductive parameters (egg volume, clutch size, number of nestlings and number of fledglings), nor the health status of the fledglings. However, in order to clarify the impact on health and reproduction a larger number of samples have to be analyzed. Phage typing showed that the isolates belonged to the definitive phage types (DT) 193 and 99, and multi-locus variable number tandem repeat analysis (MLVA) demonstrated a high similarity among the tit isolates, but distinction to human isolates. These findings suggest the presence of passerine-adapted *Salmonella* strains in free-ranging tit populations with host pathogen co-existence.

## Introduction

Infectious diseases pose an increasing threat to wildlife. Worldwide, *Salmonella* is one of the most important bacterial pathogens [1], affecting reptiles, birds and mammals [2–9]. *Salmonella enterica* subspecies *enterica* serovar Typhimurium (*Salmonella* Typhimurium) has a wide host range including humans, livestock, waterfowl, rodents and birds such as passerines [10–15].

In passerine birds, *Salmonella* Typhimurium is the most common cause of salmonellosis [3, 15, 16]. Birds can be infected through direct or indirect contact with other birds or animals, or through contact with contaminated environments [11, 15, 17–19]. Once birds are infected with this bacterium, it can be passed to their eggs during egg formation (vertical transmission) or during and after oviposition through eggshell contamination from the colonized gut or contaminated faeces (horizontal transmission) [20].

Within this serovar, the phage types DT40, DT41, DT56, and DT160 are potentially adapted to passerines and can result in endemic or context-driven epizootic infections [3, 16]. Until recently, the majority of research focused on clinical outbreaks of *Salmonella* in passerines, with clinical signs ranging from brief episodes of severe disease to acute death [11, 15, 19]. However, the poorly known and less obvious infections with host adapted strains have been suggested to have a profound impact on the birds’ reproductive success [18, 21–26]. The latter effect on host health is counterintuitive since maintenance of host-adapted pathogens in the host population would benefit from having only a minimal cost on the infected host [27, 28].

In our study, we first determined whether passerine-adapted *Salmonella* strains circulate in populations of blue tits (*Cyanistes caeruleus*) and great tits (*Parus major*), two closely related territorial hole-nesting passerines that are widely distributed throughout Europe, using molecular typing of bird-derived *Salmonella* isolates. We then correlated *Salmonella* presence on the birds’ eggs and *Salmonella* seroprevalence in fledglings with health and reproduction parameters.

## Materials and methods

### Monitoring of nest boxes of blue and great tits

**Fig 1:**
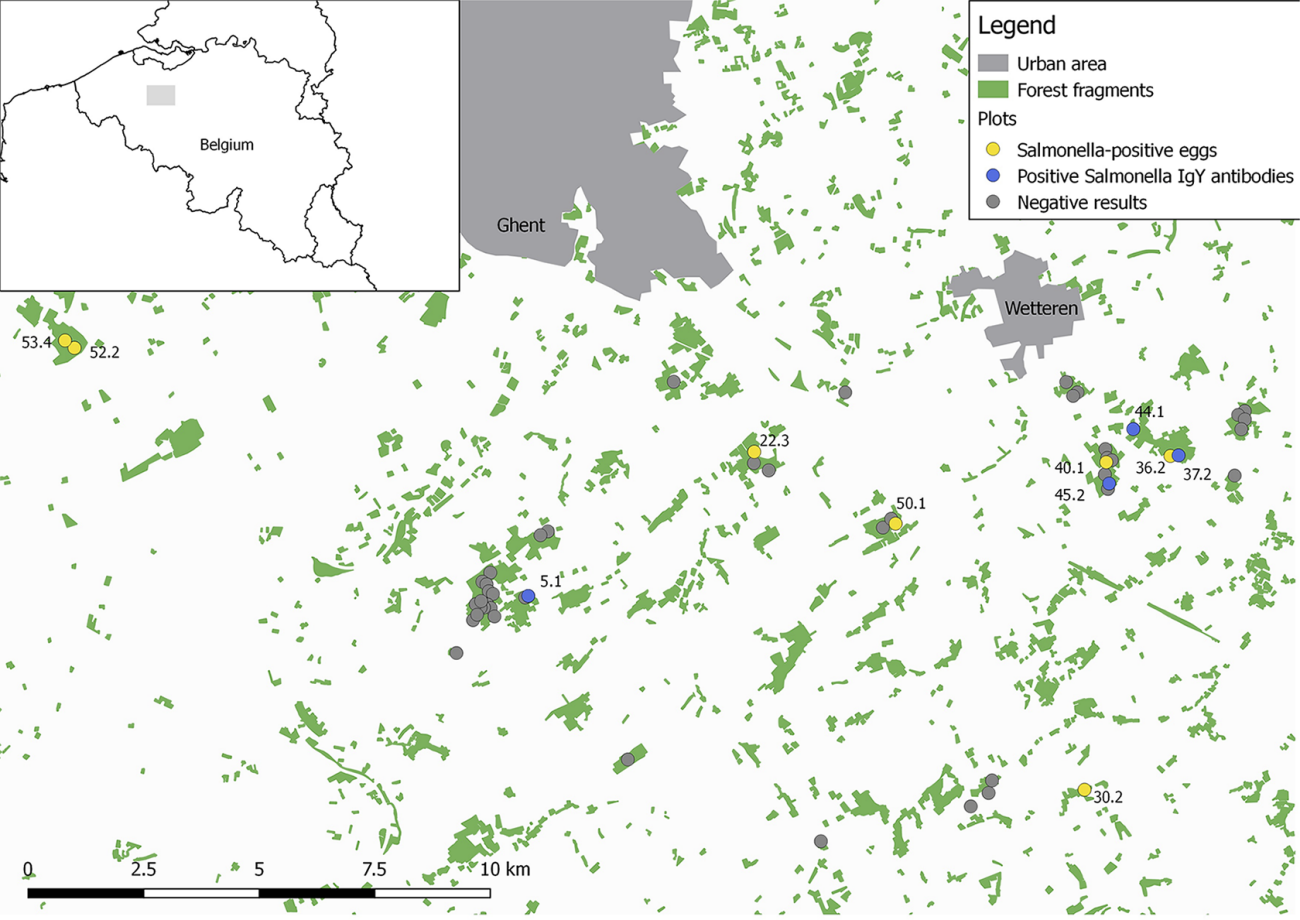
Map of the study plots showing the distribution of *Salmonella* Typhimurium. Shown are the study plots used to investigate *Salmonella* Typhimurium presence in blue and great tit nest boxes. Negative plots are indicated by grey dots, plots where *Salmonella* was found on the eggshell are represented by yellow dots and plots with nestlings carrying *Salmonella* IgY antibodies are depicted by blue dots.

In the present study, 101 eggs were sampled in 53 (30 x 30 m) study plots located in 19 mature (> 60 years) deciduous forest fragments in the south of Ghent (co: 50°57'19"N, 3°43'31"E), northern Belgium (Fig 1). These study plots (30 x 30 m) have been established to study the effects of tree species diversity and forest fragmentation on food web dynamics [29]. In the autumn of 2014, standard nest boxes for blue and great tits (dimensions 23 x 9 x 12 cm, entrance 32 mm) were installed at a height of 1.5 m, at each corner of a plot. In total, we installed 212 nest boxes of which 3 broke during the experiment. During the breeding season (April–June 2015), all nest boxes were checked twice a week to determine first-egg laying dates, then every other day to determine the laying order, the total number of eggs produced (clutch size) and the total number of nestlings and fledglings. To avoid intra-clutch variation, the fifth egg per clutch of great and blue tits was collected using sterile gloves, stored in a sterile bottle and transported to the laboratory where the eggs were cracked under a laminar flow cabinet. The egg yolk and egg white were collected. The inside of the eggshells was washed with sterile phosphate buffered saline (PBS) to remove the adhering egg albumen in order to avoid antimicrobial activity of the albumen. Bacteriological analysis of the eggshell, egg yolk and egg white was conducted as described below.

At 14–15 days of age, all fledglings were ringed and measured (tarsus (in mm) and weight (in g)). Body condition of juveniles was calculated using the scaled-mass index (SMI) [30]. Additionally, 20 μl of blood was collected from the basilic vein of 4 fledglings per nest for *Salmonella* antibody titre analysis. As blue tits are smaller than great tits, we only collected blood from great tits. All samples were kept in Eppendorf tubes at -20°C until analysis.

### Bacteriological analysis

Eggshells, including shell membranes, were transferred to an Eppendorf tube and crushed gently. Eggshell, egg yolk, and egg white samples were processed according to the ISO 6579–1:2017 method for the isolation of different *Salmonella* serovars, including *Salmonella* Typhimurium. Briefly, the samples were pre-enriched overnight in buffered peptone water at 37 ± 1°C, then enriched overnight in tetrathionate brilliant green broth (Merck, Belgium) and Rappaport Vassiliadis medium supplemented with soya (RVS) (Oxoid, UK) at 37 ± 1°C and 41.5 ± 1°C, respectively. Subsequently, the samples were plated on Brilliant Green Agar (BGA) (Oxoid, UK) and Xylose Lysine Deoxycholate (XLD) (Oxoid, UK) plates. Pink colonies on BGA or light transparent reddish with black center colonies on XLD were confirmed to be *Salmonella* based on their biochemical characteristics (glucose fermentation, H_2_S production, lysine decarboxylation positive and urea negative) [31]. All the isolates were serotyped as *Salmonella* Typhimurium using slide agglutination, targeting the antigens O4, O5 and O12. Phage typing was performed at the *Salmonella* and *Escherichia coli* reference lab of the Animal & Plant Health Agency (APHA), Weybridge, England. Multi-locus variable number tandem repeat analysis (MLVA) using the European 5-loci scheme [32] as further performed at the Scientific Institute of Public Health, Belgium [33].

### *Salmonella* antibody titre analysis

To measure IgY-anti-*Salmonella* antibodies, we applied an indirect enzyme-linked immunosorbent assay (ELISA) [26]. In summary, ELISA plates (F96 maxisorp Nunc-immuno plates, Nunc, Denmark) were coated overnight at 4°C with 140 μL of a suspension containing formalin-inactivated *Salmonella* Typhimurium DAB69 (pigeon strain) bacteria diluted in coating buffer to an optical density (OD) of 660 nm, measured with a spectrophotometer (Ultraspec III®). Each whole blood sample was thoroughly centrifuged and then diluted 1/1000 in Sample Diluent Buffer (0.6 g NaH2PO4·2H2O, 5.6 g NaH2PO4. 12H2O, 0.5 ml Tween 20 (Merck, Germany) 12.5 g NaCl, 22g skim milk powder, 1000ml distilled water) and added to the wells (100 μL) for 1 hour at 37°C. The plates were then washed three times using washing buffer (0.6 g NaH2PO4·2H2O, 5.6 g NaH2PO4. 12H2O, 0.5 ml Tween 20, 12.5 g NaCl, 1000ml distilled water). Conjugate consisting of a 1/1000 dilution of Polyclonal Goat Anti-Bird IgG (H+L)-horseradish peroxidase (HRP) conjugate (Cat-number: 90520, Alpha Diagnostics Intl. Inc., San Antonio, Texas, USA) was added and incubated at 37°C for 1 h. The plate was developed using 100 μl of 3,3′,5,5′-Tetramethylbenzidine (TMB) Liquid Substrate System for ELISA (Sigma Aldrich Chemie Gmbh, Steinheim Germany) for 15 min and stopped by the addition of 100 μl stop solution (Sigma Aldrich Chemie Gmbh, Steinheim Germany). The optical density was measured using a Multiskan MS Reader (Labsystems Oy, Helsinki, Finland) with the Ascent Software, version 2.6. All measurements were performed in duplicate.

### Statistical analysis

All statistical tests were performed with R statistical environment [34]. First, differences in *Salmonella* Typhimurium prevalence between great and blue tits were tested using a generalized linear mixed model (GLMM), R library lme4, [35]. Forest fragment identity was modeled as a random effect, species (i.e. blue versus great tit), forest fragment area size (ha), tree diversity and first-egg laying date of each clutch (Julian day) were included as fixed-effect covariates while specifying a binomial error distribution. Second, to test whether *Salmonella* Typhimurium impacts upon reproductive parameters (egg volume, clutch size, the number of nestlings, the number of fledglings and SMI of fledged young), these parameters were specified as dependent variables in linear mixed model (LMM) with as fixed effects presence or absence of *Salmonella* Typhimurium, fragment area, laying date and tree diversity. Forest fragment was again modelled as a random effect. When testing for impacts upon the number of nestlings and fledglings, clutch size was included as an additional covariate. When assessing impacts on individual fledgling SMI, we included the number of fledglings as a covariate and accounted for the non-independence of nestlings by including a nested random effect (nest box nested with forest fragment). Separate models were run for great and blue tits, and model residuals were normally distributed for all analyses (all Shapiro-Wilk W > 0.91). All continuous variables were standardized before analysis. Variable selection followed a frequentist approach whereby full models (i.e., models containing all explanatory variables considered) were reduced in a stepwise manner, by excluding the variable with the highest P-value until only P < 0.05 predictors remained. Reported statistics are derived from a minimal model (i.e. model with only the significant terms included, if any) where *Salmonella* Typhimurium presence or absence was fitted into.

### Ethical considerations

All trapping and sampling protocols were approved by the Ethical Committee VIB Ghent site (EC2015-023).

## Results

### Low *Salmonella* Typhimurium prevalence in the nests of blue and great tits

Blue and great tits only occupied nest boxes in 51 out of the 53 study plots. Great tits occupied 112 (53.59%) nest boxes and laid eggs in 66 nest boxes (31.58%). Blue tits occupied 45 nest boxes (21.53%) and eggs were found in 37 nest boxes (17.70%). The other 52 nest boxes (24.88%) remained unoccupied. In total, 65 and 36 eggs of great and blue tits, respectively, were screened for the presence of *Salmonella*. Egg contents of the screened eggs were negative for *Salmonella*. The eggshell of seven eggs (6.93% with 0–11.9 95%CI), of which four originated from blue tits, and three from great tits, were positive for *Salmonella* Typhimurium. Although relative *Salmonella* prevalence was about 2.4 times higher for blue (11.11%) compared to great tits (4.62%), these differences failed to achieve statistical significance (z-value = -0.44, P = 0.66) (Table 1 and S1 Table). The *Salmonella* Typhimurium prevalence generally is low for both tit species, however the bacterium exhibits a wide distribution between the different forests and plots (Fig 1). Six of the seven positive eggshells were found in different forests and they were all found in a different plot. Furthermore, anti-*Salmonella* antibodies were detected in four fledglings in four additional nests, in three different forest fragments and all in different plots, also suggesting a low prevalence with a wide distribution (Fig 1, Table 2, S2 Table)

**Table 1:** Reproductive parameters and SMI of blue and great tits originating from a nest containing a *Salmonella* positive eggshell.

Forest fragment	Plot	Nest Box number	Bird species	Egg volume (mm^3^)	Clutch size	n° nestlings	n° fledglings	Mean SMI ± stdev
Aelmoeseneiebos (Melle)	22	22.3	PC	7602.914	11	9	8	NC
Borsbeke (Herzele)	30	30.2	PC	5445.613	12	10	7	10.43 ± 0.66
Serskamp	36	36.2	PC	6283.4	9	8	6	11.05 ± 0.97
Oud smetlede	40	40.1	PM	10254.51	8	0	0	NA
Moortelbos (Oosterzele)	50	50.1	PC	NC	7	6	0	NA
Ooidonk (Deinze)	52	52.2	PM	10254.51	10	8	NC	NC
Ooidonk (Deinze)	53	53.4	PM	12742.74	7	NC	4	16.84 ± 0.88

Shown is the egg volume of the *Salmonella* positive eggs of blue (PC) and great (PM) tits, found in 53 analyzed plots. Per positive nest box, the clutch size, number of nestlings, number of fledglings and the brood reduction (number of nestlings–number of fledglings) are given, as well as the mean scaled-mass index (SMI) ± stdev of the nestlings. Due to practical issues, some samples were not collected (NC). If the number of fledglings was equal to 0, brood reduction and SMI could not be calculated (not applicable or NA).

**Table 2:** IgY antibody assessment in blood of great tits. Indicated are fledglings having anti-*Salmonella* antibodies (IgY) in their blood at day 14–15.

Forest fragment	Plot	Nest box number	Bird species	*Salmonella* ELISA
Heilig Geestgoed (Merelbeke)	5	5.1	PM	positive
Serskamp	37	37.2	PM	positive
Oud smetlede	44	44.1	PM	positive
Oud smetlede	45	45.2	PM	positive

### *Salmonella* Typhimurium has no effect on the reproductive fitness and SMI of blue and great tits

We first analyzed whether *Salmonella* Typhimurium affects the reproductive parameters (egg volume, clutch size, number of nestlings and fledglings) of blue and great tits. The results are summarized in Table 3, and no significant association between the presence of *Salmonella* Typhimurium on the eggshell and any reproductive parameter could be detected (Table 4). Secondly, we analyzed whether the presence of *Salmonella* Typhimurium has a negative impact on the SMI of blue and great tits. The SMI of 113 blue tit fledglings, of which eight fledglings hatched in a nest containing an egg with a *Salmonella*-positive eggshell, was calculated. In total, 186 great tit fledglings were analyzed of which four were found in a nest where we detected *Salmonella* on the eggshell. The mean SMI of blue and great tits, hatched in nest boxes where no positive eggs were found, was 11.04 ± 1.09 and 17.51 ± 1.93, respectively. The SMI in the nests containing an egg with a *Salmonella* positive eggshell, was reduced to 10.74 ± 0.84 and 16.84 ± 0.88, respectively (Table 3). However, statistical analysis showed no significant association between the presence of *Salmonella* Typhimurium on the eggshell and the SMI (Table 4).

**Table 3:** Health and reproductive parameters of blue and great tits. Shown are the mean health and reproductive parameters ± stdev, including SMI, egg volume, clutch size, number of nestlings and number of fledglings, of blue and great tits in nests containing an egg with a *Salmonella* negative or positive eggshell.

	Nests with a *Salmonella* negative eggshell		Nests with a *Salmonella* positive eggshell	
	Blue tits (n = 32)	Great tits (n = 62)	Blue tits (n = 4)	Great tits (n = 3)
SMI ± stdev	11.04 ± 1.09	17.51 ± 1.93	10.74 ± 0.84	16.84 ± 0.88
Egg volume (mm^3^) ± stdev	8226.29 ± 2223.56	10993.25 ± 2314.26	6443.98 ± 1087.58	11083.92 ± 1436.58
Clutch size ± stdev	11.03 ± 1.69	8.55 ± 1.85	9.75 ± 2.22	8.33 ± 1.53
Number of nestlings ± stdev	8.87 ± 2.31	6.42 ± 2.31	8.25 ± 1.71	4.00 ± 5.66
Number of fledglings ± stdev	7.58 ± 3.09	5.13 ± 2.77	5.25 ± 3.59	2.00 ± 2.83

**Table 4:** Statistical analysis. We investigated the association between the presence of *Salmonella* Typhimurium on the eggshell of blue and great tits and the egg volume, clutch size, number of nestlings, number of fledglings and SMI. The results are represented as the estimate ± standard deviation (stdev), degrees of freedom (d.f.), t-value and P-value.

Bird species	Association	Estimate ± stdev	d.f.	t-value	P-value
Great tits	*Salmonella* Typhimurium ~ egg volume	0.0019 ± 0.61	63	0.003	0.99
	*Salmonella* Typhimurium ~ clutch size	0.13 ± 0.58	65	-0.23	0.82
	*Salmonella* Typhimurium ~ number of nestlings	-0.97 ± 0.70	66	-1.40	0.170
	*Salmonella* Typhimurium ~ number of fledglings	-0.51 ± 0.64	63	-0.80	0.42
	*Salmonella* Typhimurium ~ SMI	0.28 ± 1.02	42	0.28	0.78
Blue tits	*Salmonella* Typhimurium ~ egg volume	-1.17 ± 1.03	22	-1.13	0.27
	*Salmonella* Typhimurium ~ clutch size	-1.35 ± 0.70	26.8	-1.93	0.064
	*Salmonella* Typhimurium ~ number of nestlings	-0.03 ± 0.49	16.5	-0.061	0.95
	*Salmonella* Typhimurium ~ number of fledglings	-0.51 ± 0.78	18.0	-0.66	0.52
	*Salmonella* Typhimurium ~ SMI	-0.85 ± 0.98	21	-0.87	0.40

### *Salmonella* Typhimurium isolates belong to bird adapted phage types DT99 and DT193

**Fig 2:**
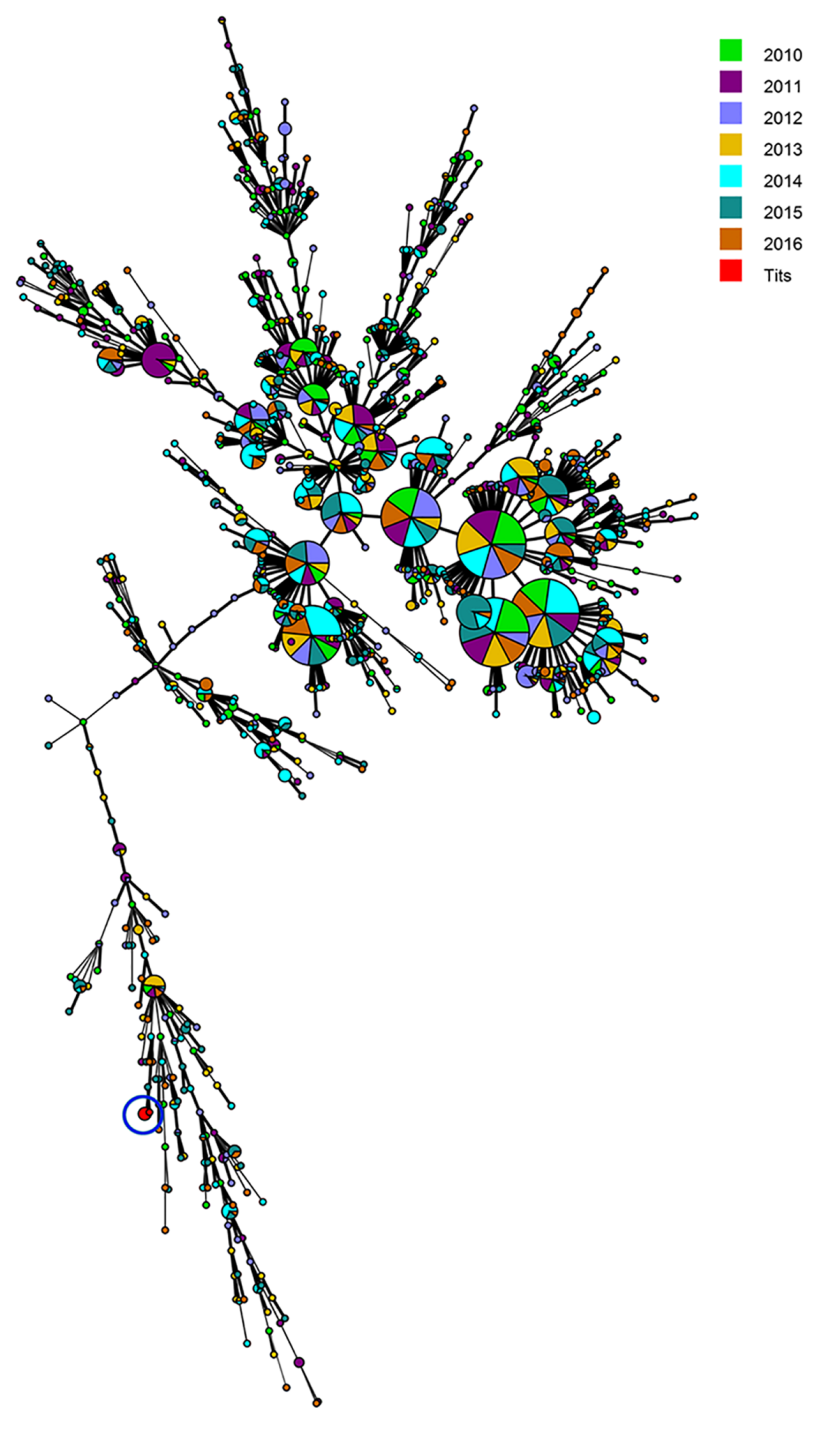
Minimum spanning tree based on MLVA data. Shown is a minimum spanning tree calculated for the MLVA profiles of the seven *Salmonella* Typhimurium isolates of blue and great tits, compared with 3239 *Salmonella* Typhimurium human isolates, regardless of phage type, in Belgium over the period of 2010–2016.

Phage typing of the seven *Salmonella* Typhimurium strains, isolated from positive eggshells (Fig 1), showed that three of them belonged to phage type DT99, whereas four belonged to phage type DT193. These isolates where further typed with MLVA, targeting five loci. Regardless of phage type, five isolates (30.2, 36.2, 50.1, 52.2, 53.4) showed identical MLVA profiles (2-16-5-13-112). For strains 22.3 and 40.1, an extra repeat was found for loci STTR5, indicating that these isolates are closely related to the other strains (Table 5). Comparing their patterns to 3239 human *Salmonella* Typhimurium isolates from 2010–2016 (database of the WIV), regardless of phage type, revealed a large distinction between the tit and human isolates (Fig 2).

**Table 5:** Phage and MLVA typing of *Salmonella* Typhimurium. Shown are the phage types and MLVA profiles of the seven *Salmonella* Typhimurium isolates targeting 5 loci.

Forest	Plot	Nest box number	bird species	Phage type	STTR9	STTR5	STTR6	STTR10	STTR3
Aelmoeseneiebos (Melle)	22	22.3	PC	DT99	2	17	5	13	0112
St-Lievens-Houtem	30	30.2	PC	DT99	2	16	5	13	0112
Serskamp	36	36.2	PC	DT99	2	16	5	13	0112
Oud smetlede	40	40.1	PM	DT193	2	17	5	13	0112
Moortelbos (Oosterzele)	50	50.1	PC	DT193	2	16	5	13	0112
Ooidonk (Deinze)	52	52.2	PM	DT193	2	16	5	13	0112
Ooidonk (Deinze)	53	53.4	PM	DT193	2	16	5	13	0112

## Discussion

This study found a broad distribution at a low prevalence (± 7%) of two *Salmonella* Typhimurium phage types (DT99 and DT193) in populations of apparently healthy great and blue tits in Flanders, Belgium. The low prevalence was also confirmed by the seroprevalence of the fledglings. Surprisingly, none of the fledglings originating from a nest where we detected *Salmonella* on the eggshell was seropositive and conversely, all *Salmonella* seropositive fledglings were not from a nest containing a positive eggshell. This could have different reasons. Firstly, it is possible that the isolated strains have a limited chance of trans-shell infection, explaining the fact that egg yolk and white were negative for *Salmonella*. Secondly, nestlings became infected after hatching. This could occur through contact with contaminated nest material, which we did not screen for the presence of *Salmonella*. Thirdly, as we only screened one egg per nest, it possible that we missed other positive eggs in the nest, or that the other eggs in the nest were negative. Therefore, it is possible that our results are an underestimation of the *Salmonella* prevalence. Fourth, it is possible that the detected antibodies are maternal antibodies from an earlier infection. In chickens, around 3–4 days after hatching the juveniles begin the synthesize their own antibodies, however maternal antibodies can persist in the chick's circulation for 14 days after hatching [36]. Therefore, it is possible that the maternal immunity biased the serological results.

DT99 is usually considered a pigeon-adapted variant of *Salmonella* Typhimurium, which circulates endemically in feral pigeons in Belgium [37] but has also been associated with mortality in passerines [26, 38]. Infections can cause systemic disease associated with high mortality rates in pigeons [14]. Clinical manifestations include gastroenteritis, arthritis, oophoritis or orchitis and systemic granulomatous inflammation [39]. Feral pigeons may serve as a source of infection for passerines especially in high aggregation areas such as bird feeding stations. DT193 is commonly associated with human infections [33, 40–44]. Additionally, this phage type has also been associated with disease outbreaks in birds on a few occasions [3]. Wild birds are considered as carriers of *Salmonella*, causing salmonellosis in both humans and domestics animals [10, 13]. The MLVA typing of both tit phage types in comparison with human isolates of *Salmonella* Typhimurium showed a high level of genetic similarity between the different tit isolates, but a large distinction between human and tit isolates. It is therefore possible that the isolates belonging to DT193 in this study represent avian-adapted *Salmonella* Typhimurium strains in free-ranging tits. If so, these *Salmonella* Typhimurium tit isolates possibly have a low impact on humans. However, more epidemiological data are needed to confirm the host range and to support our hypothesis. Furthermore, our data cannot be generalized as nest location and contact with human-made environment are important factors that can influence the epidemiology of specific *Salmonella* strains [3, 4]. Possibly, providing nest location in the surrounding of human settlements could lead to the isolation of *Salmonella* isolates with an MLVA profile closely linked to human isolates and with zoonotic and epizootic potential. Taking this in account, it is very likely that the distinction that we observe between the human and tit isolates is related to the sampling location. Therefore, it would be interesting to investigate the difference in *Salmonella* presence and their impact in both rural and urban forests.

Although pathogen persistence in specific host populations is an essential mechanism of host-adapted pathogens [45], costs and benefits for the host population during a state of pathogen endemism have been poorly studied. Host adaptation has been associated with systemic disease and increased severity of infection [46, 47]. On the contrary, hosts can benefit from host–pathogen coevolution, as it can lead to a lower pathogenicity and mortality [27, 28]. We did not observe any health or reproduction-related impacts from the presence of *Salmonella* Typhimurium on the eggshells. This finding is in line with the hypothesis that a limited impact of pathogen burden on host health allows host-pathogen co-existence and pathogen population maintenance in its primary niche, the host. However, the limited number of *Salmonella* positive nests in our study raises the need for extra experimental or field studies with a much bigger positive sample size.

In summary, our results indicate that *Salmonella* Typhimurium is present in free-ranging tit populations, without representing a major risk for reproductive success and health status. It is possible that, by limiting the impact of the pathogen burden on host health, *Salmonella* is able to persist and establish a wide distribution pattern. Although there is limited evidence that these strains currently have epizootic and/or zoonotic potential, we cannot state that free-ranging tits cannot transmit *Salmonella* Typhimurium to humans and other non-human animals. Since the host-pathogen interaction is driven by host characteristics, pathogen virulence and environmental drivers, including static and dynamic pathogen reservoirs, changes in any of these compounds of the disease triangle [48] may shift the state of co-existence towards the epizootics that have been described before [16, 49]. Future studies on the drivers of infection and disease dynamics are thus vital to understand the impact of *Salmonella* infections in wild birds.

## Supporting information

S1 Table Overview of the *Salmonella* status, reproductive parameters and SMI in nests of blue and great tits. Shown are the nests with eggs of blue (PC) and great (PM) tits in 53 different plots. Every 5^th^ egg was weighted (volume) and bacteriologically analyzed for the presence of *Salmonella* (negative or positive). Per nest box, the clutch size, number of nestlings and number of fledglings are given, as well as the mean scaled-mass index (SMI) ± stdev of the nestlings. Due to practical issues, some samples were not collected (NC). If the number of fledglings was equal to 0, brood reduction and SMI could not be calculated (not applicable or NA). An asterisk (*) indicates a nest box that was occupied twice by both PC and PM.
(DOCX)

S2 Table IgY antibody assessment in blood of great tits. Using a *Salmonella* specific ELISA, as described in the materials and methods section, the presence of IgY antibodies in the blood of blue tits (PM) was analyzed as negative or positive. Due to practical issues, some samples were not collected (NC). If the number of fledglings was equal to 0, no blood could be taken (not applicable or NA).
(DOCX)
