# Supplementary material for: Salmonella Typhimurium DT193 and DT99 are present in great and blue tits in Flanders, Belgium
Source: PLoS One. 2017 Nov 7;12(11):e0187640. doi: 10.1371/journal.pone.0187640 (PMC5675436; doi:10.1371/journal.pone.0187640)
Supplement: S1 Table — Shown are the nests with eggs of blue (PC) and great (PM) tits in 53 different plots. Every 5th egg was weighted (volume) and bacteriologically analyzed for the presence of Salmonella (negative or positive). Per nest box, the clutch size, number of nestlings and number of fledglings are given, as well as the mean scaled-mass index (SMI) ± stdev of the nestlings. Due to practical issues, some samples were not collected (NC). If the number of fledglings was equal to 0, brood reduction and SMI could not be calculated (not applicable or NA). An asterisk (*) indicates a nest box that was occupied twice by both PC and PM. (DOCX) [file pone.0187640.s001.docx]

**Supplementary Table 1: Overview of the *Salmonella* status, reproductive parameters and SMI in nests of blue and great tits.** Shown are the nests with eggs of blue (PC) and great (PM) tits in 53 different plots. Every 5^th^ egg was weighted (volume) and bacteriologically analyzed for the presence of *Salmonella* (negative or positive). Per nest box, the clutch size, number of nestlings and number of fledglings are given, as well as the mean scaled-mass index (SMI) ± stdev of the nestlings. Due to practical issues, some samples were not collected (NC). If the number of fledglings was equal to 0, brood reduction and SMI could not be calculated (not applicable or NA). An asterisk (*) indicates a nest box that was occupied twice by both PC and PM.

| **Forest fragment** | **Forest fragment surface area (ha )** | **Plot** | **Nest**  **box** | **Spp** | ***Salmonella*status** | **Egg volume (mm^3^)** | **Clutch size** | **N° nestlings** | **N° fledglings** | **Mean SMI ± stdev** |
| --- | --- | --- | --- | --- | --- | --- | --- | --- | --- | --- |
| Vurtzak (Merelbeke) | 16.68 | 1 | 1.2 | PM | negative | 10254.51 | 8 | 7 | 7 | 15.78 ± 0.15 |
|  |  |  | 1.3 | PC | negative | NC | 11 | 0 | 0 | NA |
|  |  |  | 1.4 | PM | negative | 12034.81 | 9 | 8 | 8 | NC |
| Nerenbos (Merelbeke) | 41.74 | 2 | 2.1 | PC | negative | 7602.91 | 11 | 10 | 9 | 11.60 ± 0.70 |
|  |  |  | 2.2 | PC | negative | NC | 8 | 7 | 7 | 11.79 ± 3.33 |
|  |  |  | 2.3 | PM | negative | 12742.74 | 8 | 5 | 5 | 18.03 ± 0.62 |
|  |  |  | 2.4 | PM | negative | 9651.30 | 8 | 4 | 4 | 16.76 ± 1.13 |
|  |  | 3 | 3.2 | PM | negative | 7602.91 | 5 | 4 | 4 | 18.55 ± 1.20 |
|  |  |  | 3.4 | PC | negative | 15599.59 | 10 | 7 | 4 | 10.45 ± 1.15 |
| Heilig Geestgoed (Merelbeke) | 27.49 | 4 | 4.1 | PM | negative | 12034.81 | 8 | 5 | 5 | 20.29 ± 2.70 |
|  |  |  | 4.3 | PM | negative | 11326.88 | 10 | 7 | 0 | NA |
|  |  | 5 | 5.1 | PM | negative | 8109.77 | 7 | 2 | 2 | 17.83 ± 1.29 |
|  |  |  | 5.2 | PM | negative | 10254.51 | 6 | 4 | 4 | 16.36 ± 0.52 |
| Makegembos (Merelbeke) | 83.77 | 7 | 7.1 | PM | negative | 12742.74 | 9 | 7 | 3 | 13.33 ± 1.00 |
|  |  |  | 7.2 | PC | negative | 6283.40 | 10 | 9 | 9 | 11.83 ± 0.40 |
|  |  |  | 7.3 | PM | negative | 11460.92 | 9 | 8 | 8 | 17.15 ± 0.89 |
|  |  | 8 | 8.2 | PC | negative | 5864.51 | 10 | 9 | 9 | 9.49 ± 0.70 |
|  |  | 9 | 9.1 | PC | negative | 8109.77 | 10 | 9 | 9 | NC |
|  |  |  | 9.3 | PM | negative | 16965.18 | 8 | 7 | 7 | 16 54 ± 0.39 |
|  |  |  | 9.4 | PM | negative | 10857.72 | 7 | 5 | 5 | 16.97 ± 0.93 |
|  |  | 10 | 10.3 | PM | negative | 10857.72 | 10 | 6 | 6 | NC |
| Harentbeekbos (Merelbeke) | 83.77 | 12 | 12.1 | PC | negative | 7602.91 | 8 | 6 | 5 | 11.98 ± 0.56 |
|  |  |  | 12.3 | PM | negative | 12742.74 | 9 | 7 | 7 | 17.62 ± 1.17 |
|  |  |  | 12..4 | PM | negative | 12034.81 | 9 | 8 | 8 | 15.97 ± 0.39 |
|  |  | 13 | 13.2 | PC | negative | 8109.77 | 11 | 10 | 10 | 11.71 ± 0.51 |
|  |  |  | 13.3 | PC* | negative | 7602.91 | 11 | 9 | 8 | 12.71 ± 2.36 |
|  |  |  |  | PM* | NC | NC |  |  |  | 14.88 ± 0.39 |
|  |  | 16 | 16.1 | PM | negative | 9630.36 | 6 | 5 | 4 | 16.59 ± 0.31 |
|  |  |  | 16.2 | PC | negative | 7602.91 | 9 | 7 | 7 | 10.74 ± 0.94 |
|  |  |  | 16.4 | PM | negative | 10857.72 | 8 | 7 | 5 | 16.57 ± 0.76 |
|  |  | 17 | 17.1 | PC | negative | 6283.40 | 14 | 13 | 13 | 9.83 ± 0.36 |
|  |  |  | 17.2 | PM | negative | 10857.72 | 8 | 6 | 6 | 16.90 ± 0.82 |
|  |  |  | 17.3 | PC | negative | 14778.56 | 12 | 10 | 9 | 10.80 ± 0.51 |
|  |  | 18 | 18.1 | PM | negative | 10254.51 | 11 | 10 | 10 | 18.75 ± 1.80 |
|  |  |  | 18.2 | PM | negative | 9123.50 | 8 | 6 | 6 | 16.89 ± 1.00 |
|  |  |  | 18.3 | PM | negative | 11326.88 | 8 | 5 | 5 | NC |
|  |  |  | 18.4 | PM | negative | 10254.51 | 10 | 7 | 7 | 17.11 ± 1.09 |
|  |  | 19 | 19.3 | PM | negative | 6283.40 | 9 | 8 | 8 | 16.99 ± 0.66 |
|  |  |  | 19.4 | PC | negative | 7841.68 | 14 | 9 | 9 | 11.30 ± 0.68 |
| Wannegatstrt (Gavere) | 3.03 | 20 | 20.1 | PM | negative | 12742.74 | 13 | 11 | 9 | NC |
|  |  |  | 20.2 | PC | negative | 7602.91 | 11 | 9 | 7 | NC |
|  |  |  | 20.3 | PC | negative | 6283.40 | 13 | 11 | 0 | NA |
| Bueren (Melle) | 6.19 | 21 | 21.1 | PC | negative | 8109.77 | 12 | 9 | 9 | 10.41 ± 0.56 |
|  |  |  | 21.2 | PM | negative | 10254.51 | 7 | 5 | 5 | 17.40 ± 0.89 |
| Aelmoeseneiebos (Melle) | 23.57 | 22 | 22.1 | PM | negative | 13450.66 | 8 | 6 | 6 | NC |
|  |  |  | 22.2 | PM | negative | 14778.56 | 6 | 4 | 0 | NA |
|  |  |  | **22.3** | **PC** | **positive** | 7602.91 | **11** | **9** | **8** | **NC** |
|  |  |  | 22.4 | PC | negative | 8109.77 | 11 | 8 | 7 | 9.52 ± 1.87 |
|  |  | 23 | 23.1 | PM | negative | 6283.40 | 8 | 7 | 6 | 23.57 ± 1.57 |
|  |  |  | 23.3 | PM | negative | 6283.40 | 8 | 5 | 5 | 22.25 ± 1.56 |
|  |  | 24 | 24.1 | PM | negative | 10254.51 | 6 | 5 | 5 | NC |
|  |  |  | 24.2 | PM | negative | 6702.29 | 9 | 8 | 0 | NA |
|  |  |  | 24.4 | PM | negative | 12742.74 | 10 | 9 | 7 | NC |
| Spiegeldries bos (Oosterzele) | 11.37 | 25 | 25.1 | PM | negative | 12742.74 | 10 | 9 | 8 | 18.72 ± 1.00 |
|  |  |  | 25.2 | PM | negative | 9048.10 | 10 | 9 | 7 | 17.78 ± 0.76 |
|  |  |  | 25.3 | PM | negative | 10254.51 | 9 | 0 | 0 | NA |
| St-Lievens-Houtem | 1.31 | 27 | 27.4 | PM | negative | 12034.81 | 9 | 4 | 0 | NA |
|  | 1.59 | 28 | 28.2 | PC | negative | 8109.77 | 12 | 9 | 7 | NC |
|  |  |  | 28.3 | PC | negative | 6283.40 | NC | NC | NC | NC |
|  |  |  | 28.4 | PM | negative | 10254.51 | 8 | 6 | 5 | 18.02 ± 0.70 |
|  | 5.63 | 29 | 29.2 | PC | negative | NC | 10 | 9 | 9 | 11.80 ± 1.47 |
|  |  |  | 29.4 | PM | negative | 10254.51 | 10 | 9 | 9 | 17.29 ± 0.65 |
| Borsbeke (Herzele) | 12.04 | 30 | 30.1 | PC | negative | 8109.77 | 13 | 8 | 5 | 11.69 ± 0.56 |
|  |  |  | **30.2** | **PC** | **positive** | 5445.61 | **12** | **10** | **7** | **10.43 ± 0.66** |
|  |  |  | 30.3 | PM | negative | 15599.59 | 11 | 7 | 5 | NC |
|  |  |  | 30.4 | PC | negative | NC | 12 | 11 | 11 | 10. 98 ± 0.47 |
|  | 9.21 | 31 | 31.3 | PM | negative | 8109.77 | 8 | 7 | 7 | 18.23 ± 1.83 |
| Nonnenbos (Serskamp) | 32.69 | 32 | 32.2 | PM | negative | 6283.40 | 11 | 9 | 0 | NA |
| Serskamp | **58.90** | **36** | **36.2** | **PC** | **positive** | 6283.40 | **9** | **8** | **6** | **11.05 ± 0.97** |
|  |  |  | 36.4 | PC | negative | 8109.77 | 7 | 6 | 5 | 11.53 ± 0.13 |
|  |  | 37 | 37.2 | PM | negative | 13450.66 | 9 | 7 | 2 | 14.96 ± 0.33 |
|  |  |  | 37.3 | PM | negative | 10254.51 | 7 | 6 | 3 | 17.10 ± 1.78 |
|  |  |  | 37.4 | PM | negative | 10857.72 | 8 | 5 | 3 | 17.52 ± 1.02 |
| Oud smetledebos | 47.77 | 38 | 38.1 | PM | negative | 11326.88 | 11 | 10 | 10 | 16.50 ± 0.58 |
| (Smetlede) |  |  | 38.3 | PM | negative | 12742.74 | 6 | 4 | 4 | 18.33 ± 1.08 |
|  |  |  | 38.4 | PM | negative | 10254.51 | 8 | 7 | 7 | 17.95 ± 2.20 |
|  |  | 39 | 39.4 | PM | negative | 13957.53 | 7 | 6 | 0 | NA |
|  |  | **40** | **40.1** | **PM** | **positive** | 10254.51 | **8** | **0** | **0** | **NA** |
|  |  | 41 | 41.3 | PM | negative | NC | 8 | 7 | 6 | 17. 72 ± 0.79 |
|  |  | 43 | 43.1 | PC | negative | 8109.77 | 12 | 10 | 10 | 11.47 ± 0.61 |
|  |  |  | 43.2 | PM | negative | 12742.74 | 8 | 7 | 7 | 17.89 ± 0.85 |
|  |  | 44 | 44.1 | PM | negative | 12742.74 | 8 | 7 | 6 | 17.36 ± 0.67 |
|  | 58.90 | 45 | 45.2 | PM* | negative | 7602.91 | 17 | 14 | 10 | 14.66 ± 0.82 |
|  |  |  |  | PC* | NC | NC |  |  |  | 8.77 ± 1.22 |
| Hospiesbos (Wetteren) | 18.73 | 46 | 46.3 | PC | negative | 8109.77 | 11 | 10 | 9 | 12.34 ± 0.27 |
|  |  | 47 | 47.1 | PM | negative | NC | 7 | 5 | 5 | 17.36 ± 0.69 |
|  |  |  | 47.2 | PM | negative | 10254.51 | 8 | 5 | 5 | 17.30 ± 2.42 |
|  |  | 48 | 48.1 | PC | negative | NC | 10 | 9 | 9 | 10.48 ± 1.18 |
|  |  |  | 48.2 | PM | negative | 12742.74 | 8 | 6 | 6 | 18.12 ± 0.15 |
|  |  |  | 48.4 | PM | negative | 12742.74 | 8 | 7 | 0 | NA |
| Moortelbos (Oosterzele) | 30.65 | 49 | 49.1 | PM | negative | 7602.91 | 6 | 5 | 5 | 18.63 ± 1.21 |
|  |  |  | 49.3 | PM | negative | 12742.74 | 8 | 6 | 6 | 16.49 ± 0.87 |
|  |  |  | 49.4 | PC | negative | 10618.95 | 12 | 9 | 9 | 11.51 ± 0.41 |
|  |  | **50** | **50.1** | **PC** | **positive** | NC | **7** | **6** | **0** | **NA** |
|  |  |  | 50.2 | PM | negative | 12742.74 | 10 | 9 | 8 | 16.52 ± 0.60 |
|  |  |  | 50.3 | PC | negative | 6702.29 | 13 | 12 | 0 | NA |
|  |  |  | 50.4 | PM | negative | 12742.74 | 10 | 0 | 0 | NA |
|  |  | 51 | 51.1 | PM | negative | 10254.51 | 9 | 7 | 7 | 16.60 ± 0.77 |
|  |  |  | 51.2 | PC | negative | 7841.68 | 12 | 10 | 10 | NC |
|  |  |  | 51.4 | PC | negative | 8109.77 | 9 | 9 | 9 | 11.02 ± 0.74 |
| Ooidonk (Deinze) | **46.16** | **52** | **52.2** | **PM** | **positive** | 10254.51 | **10** | **8** | **NC** | **NC** |
|  |  | 53 | 53.1 | PC | negative | 8616.64 | 13 | 11 | 11 | 11.12 ± 1.07 |
|  |  |  | **53.4** | **PM** | **positive** | 12742.74 | **7** | **NC** | **4** | **16.84 ± 1.01** |
