# Supplementary material for: Salmonella Typhimurium DT193 and DT99 are present in great and blue tits in Flanders, Belgium
Source: PLoS One. 2017 Nov 7;12(11):e0187640. doi: 10.1371/journal.pone.0187640 (PMC5675436; doi:10.1371/journal.pone.0187640)
Supplement: S2 Table — Using a Salmonella specific ELISA, as described in the materials and methods section, the presence of IgY antibodies in the blood of blue tits (PM) was analyzed as negative or positive. Due to practical issues, some samples were not collected (NC). If the number of fledglings was equal to 0, no blood could be taken (not applicable or NA). (DOCX) [file pone.0187640.s002.docx]

**Supplementary Table 2: IgY antibody assessment in blood of great tits.** Using a *Salmonella* specific ELISA, as described in the materials and methods section, the presence of IgY antibodies in the blood of blue tits (PM) was analyzed as negative or positive. Due to practical issues, some samples were not collected (NC). If the number of fledglings was equal to 0, no blood could be taken (not applicable or NA).

| **Forest** | **Plot** | **Nestbox** | **Spp.** | **Juvenile** | ***Salmonella* ELISA** |
| --- | --- | --- | --- | --- | --- |
| Vurtzak (Merelbeke) |  | 1.2 | PM | 58V92692 | negative |
|  |  |  |  | 58V92693 | negative |
|  |  |  |  | 58V92694 | negative |
|  |  |  |  | 58V92698 | NC |
|  |  | 1.4 | PM | 58V92930 | negative |
|  |  |  |  | 58V92932 | negative |
|  |  |  |  | 58V92933 | negative |
| Nerenbos (Merelbeke) | 2 | 2.3 | PM | 58V92852 | negative |
|  |  |  |  | 58V92853 | negative |
|  |  |  |  | 58V92854 | negative |
|  |  |  |  | 58V92855 | NC |
|  |  |  |  | 58V92966 | negative |
|  |  | 2.4 | PM | 58V92857 | negative |
|  |  |  |  | 58V92858 | negative |
|  |  |  |  | 58V92859 | NC |
|  | 3 | 3.2 | PM | 58V92861 | negative |
|  |  |  |  | 58V92862 | negative |
|  |  |  |  | 58V92863 | negative |
|  |  |  |  | 58V92864 | NC |
| Heilig Geestgoed (Merelbeke) | 4 | 4.1 | PM | 58V92812 | negative |
|  |  |  |  | 58V92813 | NC |
|  |  |  |  | 58V92814 | negative |
|  |  |  |  | 58V92815 | negative |
|  |  |  |  | 58V92816 | NC |
|  |  | 4.3 | PM | NA | NA |
|  | 5 | 5.1 | PM | 58V92796 | negative |
|  |  |  |  | **58V92797** | **positive** |
|  |  | 5.2 | PM | 58V92601 | NC |
|  |  |  |  | 58V92602 | negative |
|  |  |  |  | 58V92798 | negative |
|  |  |  |  | 58V92799 | negative |
| Makegembos (Merelbeke) | 7 | 7.1 | PM | 58V92545 | negative |
|  |  |  |  | 58V92546 | NC |
|  |  |  |  | 58V92547 | NC |
|  |  | 7.3 | PM | 58V92537 | negative |
|  |  |  |  | 58V92538 | negative |
|  |  |  |  | 58V92539 | negative |
|  |  |  |  | 58V92541 | negative |
|  | 9 | 9.3 | PM | 58V92605 | negative |
|  |  |  |  | 58V92607 | negative |
|  |  |  |  | 58V92609 | negative |
|  |  |  |  | 58V92611 | negative |
|  |  | 9.4 | PM | 58V92549 | negative |
|  |  |  |  | 58V92550 | negative |
|  |  |  |  | 58V92551 | negative |
|  |  |  |  | 58V92552 | NC |
|  | 10 | 10.3 | PM | NC | NC |
| Harentbeekbos (Merelbeke) | 12 | 12.3 | PM | 58V92866 | negative |
|  |  |  |  | 58V92867 | negative |
|  |  |  |  | 58V92868 | negative |
|  |  |  |  | 58V92869 | negative |
|  |  | 12..4 | PM | 57V84782 | negative |
|  |  |  |  | 57V84783 | negative |
|  |  |  |  | 57V84784 | negative |
|  |  |  |  | 57V84785 | negative |
|  | 13 | 13.3 | PM | 58V92581 | NC |
|  |  |  |  | 58V92583 | negative |
|  |  |  |  | 58V92584 | NC |
|  |  |  |  | 58V92586 | negative |
|  | 16 | 16.1 | PM | 58V92615 | negative |
|  |  |  |  | 58V92616 | negative |
|  |  |  |  | 58V92617 | negative |
|  |  |  |  | 58V92618 | negative |
|  |  | 16.4 | PM | 58V92620 | negative |
|  |  |  |  | 58V92621 | negative |
|  |  |  |  | 58V92622 | NC |
|  |  |  |  | 58V92624 | negative |
|  | 17 | 17.2 | PM | 58V92628 | negative |
|  |  |  |  | 58V92629 | negative |
|  |  |  |  | 58V92630 | negative |
|  |  |  |  | 58V92633 | NC |
|  | 18 | 18.1 | PM | 58V92873 | negative |
|  |  |  |  | 58V92874 | negative |
|  |  |  |  | 58V92875 | negative |
|  |  |  |  | 58V92876 | negative |
|  |  |  |  | 58V92877 | negative |
|  |  |  |  | 58V92878 | negative |
|  |  |  |  | 58V92879 | negative |
|  |  |  |  | 58V92880 | negative |
|  |  | 18.2 | PM | 58V92642 | negative |
|  |  |  |  | 58V92643 | negative |
|  |  |  |  | 58V92644 | NC |
|  |  |  |  | 58V92645 | negative |
|  |  | 18.3 | PM | NC | NC |
|  |  | 18.4 | PM | 57V84790 | NC |
|  |  |  |  | 57V84791 | negative |
|  |  |  |  | 57V84792 | negative |
|  |  |  |  | 57V84793 | negative |
|  | 19 | 19.3 | PM | 58V92634 | negative |
|  |  |  |  | 58V92635 | negative |
|  |  |  |  | 58V92638 | NC |
|  |  |  |  | 58V92639 | negative |
| Wannegatstraat (Gavere) | 20 | 20.1 | PM | NC | NC |
| Bueren (Melle) | 21 | 21.2 | PM | 58V92671 | negative |
|  |  |  |  | 58V92672 | negative |
|  |  |  |  | 58V92674 | negative |
|  |  |  |  | 58V92675 | negative |
| Aelmoeseneiebos (Melle) | 22 | 22.1 | PM | NC | NC |
|  |  | 22.2 | PM | NA | NA |
|  | 23 | 23.1 | PM | 58V92656 | negative |
|  |  |  |  | 58V92657 | NC |
|  |  |  |  | 58V92660 | negative |
|  |  |  |  | 58V92662 | negative |
|  |  | 23.3 | PM | 58V92663 | negative |
|  |  |  |  | 58V92664 | negative |
|  |  |  |  | 58V92667 | negative |
|  |  |  |  | 58V92668 | NC |
|  |  |  |  | 58V92669 | negative |
|  | 24 | 24.1 | PM | NC | NC |
|  |  | 24.2 | PM | NA | NA |
|  |  | 24.4 | PM | NC | NC |
| Spiegeldriesbos (Oosterzele) | 25 | 25.1 | PM | 58V84797 | negative |
|  |  |  |  | 58V84798 | negative |
|  |  |  |  | 58V84799 | NC |
|  |  |  |  | 57V84800 | negative |
|  |  | 25.2 | PM | 58V92805 | NC |
|  |  |  |  | 58V92806 | NC |
|  |  |  |  | 58V92807 | NC |
|  |  |  |  | 58V92808 | NC |
|  |  | 25.3 | PM | NA | NA |
| St-Lievens-Houtem | 27 | 27.4 | PM | NA | NA |
|  | 28 | 28.4 | PM | 58V92990 | negative |
|  |  |  |  | 58V92991 | NC |
|  |  |  |  | 58V92992 | negative |
|  |  |  |  | 58V92993 | negative |
|  | 29 | 29.4 | PM | 58V92701 | negative |
|  |  |  |  | 58V92704 | negative |
|  |  |  |  | 58V92997 | negative |
|  |  |  |  | 58V92998 | negative |
| Borsbeke (Herzele) | 30 | 30.3 | PM | 58V92766 | negative |
|  |  |  |  | 58V92768 | negative |
|  | 31 | 31.3 | PM | 58V92742 | negative |
|  |  |  |  | 58V92744 | negative |
|  |  |  |  | 58V92745 | negative |
| Nonnenbos (Serskamp) | 32 | 32.2 | PM | NA | NA |
| Serskamp | 37 | 37.2 | PM | 58V92504 | NC |
|  |  |  |  | 58V92505 | negative |
|  |  |  |  | 58V92506 | NC |
|  |  |  |  | **58V92507** | **positive** |
|  |  | 37.3 | PM | 58V92501 | negative |
|  |  |  |  | 58V92502 | NC |
|  |  |  |  | 58V92503 | negative |
|  |  |  |  | 58V92900 | negative |
|  |  | 37.4 | PM | 58V94773 | negative |
|  |  |  |  | 58V94774 | negative |
| Oud smetledebos (Smetlede) | 38 | 38.1 | PM | 57V84756 | NC |
|  |  |  |  | 57V84757 | NC |
|  |  |  |  | 57V84758 | NC |
|  |  |  |  | 57V84759 | NC |
|  |  | 38.3 | PM | 58V92758 | negative |
|  |  |  |  | 58V92759 | negative |
|  |  |  |  | 58V92760 | NC |
|  |  |  |  | 58V92761 | negative |
|  |  | 38.4 | PM | 57V84766 | NC |
|  |  |  |  | 57V84767 | NC |
|  |  |  |  | 57V84768 | NC |
|  | 39 | 39.4 | PM | 58V92883 | NC |
|  |  |  |  | 58V92884 | negative |
|  | 40 | 40.1 | PM | NA | NA |
|  | 41 | 41.3 | PM | 58V92787 | negative |
|  |  |  |  | 58V92788 | negative |
|  |  |  |  | 58V92789 | negative |
|  |  |  |  | 58V92790 | negative |
|  | 43 | 43.2 | PM | 58V92763 | negative |
|  |  |  |  | 58V92764 | NC |
|  |  |  |  | 58V92765 | NC |
|  |  |  |  | 58V92766 | NC |
|  |  |  |  | 58V92776 | negative |
|  |  |  |  | 58V92898 | NC |
|  |  |  |  | 58V92899 | negative |
|  | 44 | 44.1 | PM | 58V92777 | negative |
|  |  |  |  | 58V92778 | negative |
|  |  |  |  | 58V92779 | negative |
|  |  |  |  | **58V92781** | **positive** |
|  | 45 | 45.2 | PM | 58V92571 | NC |
|  |  |  |  | 58V92572 | negative |
|  |  |  |  | **58V92575** | **positive** |
|  |  |  |  | 58V92577 | negative |
| Hospiesbos (Wetteren) | 47 | 47.1 | PM | 58V92710 | negative |
|  |  |  |  | 58V92711 | negative |
|  |  |  |  | 58V92712 | negative |
|  |  |  |  | 58V92713 | negative |
|  |  | 47.2 | PM | 58V92733 | negative |
|  |  |  |  | 58V92734 | negative |
|  |  |  |  | 58V92736 | negative |
|  |  |  |  | 58V92737 | NC |
|  | 48 | 48.2 | PM | 58V92683 | negative |
|  |  |  |  | 58V92685 | negative |
|  |  |  |  | 58V92687 | negative |
|  |  |  |  | 58V92688 | negative |
|  |  | 48.4 | PM | NA | NA |
| Moortelbos (Oosterzele) | 49 | 49.1 | PM | 58V92820 | NC |
|  |  |  |  | 58V92821 | negative |
|  |  |  |  | 58V92822 | negative |
|  |  |  |  | 58V92823 | negative |
|  |  | 49.3 | PM | 58V92564 | negative |
|  |  |  |  | 58V92565 | NC |
|  |  |  |  | 58V92566 | negative |
|  |  |  |  | 58V92568 | NC |
|  | 50 | 50.2 | PM | 58V92532 | negative |
|  |  |  |  | 58V92533 | NC |
|  |  |  |  | 58V92535 | negative |
|  |  | 50.4 | PM | NA | NA |
|  | 51 | 51.1 | PM | 58V92827 | negative |
|  |  |  |  | 58V92828 | NC |
|  |  |  |  | 58V92829 | negative |
|  |  |  |  | 58V92830 | negative |
| Ooidonk (Deinze) | 52 | 52.2 | PM | NC | NC |
|  | 53 | 53.4 | PM | 58V92983 | NC |
|  |  |  |  | 58V92984 | negative |
|  |  |  |  | 58V92985 | negative |
|  |  |  |  | 58V92986 | negative |
